# Acetyl­ferrocene–2-chloro-1-ferrocenyl­ethanone (1/1)

**DOI:** 10.1107/S1600536811038244

**Published:** 2011-09-30

**Authors:** Milan Erben, Jaromír Vinklárek, Aleš Růžička

**Affiliations:** a Department of General and Inorganic Chemistry, Faculty of Chemical Technology, University of Pardubice, Studentská 95, Pardubice 532 10, Czech Republic

## Abstract

In the title co-crystal, [Fe(C_5_H_5_)(C_7_H_6_ClO)][Fe(C_5_H_5_)(C_7_H_7_O)], both substituted ferrocene mol­ecules show the expected sandwich structure. The crystal packing exhibits weak inter­molecular Cl⋯Cl contacts of 3.279 (4) Å, π–π inter­actions between the substituted Cp rings of two neighbouring 2-chloro-1-ferrocenyl­ethanone mol­ecules [centroid–centroid distance = 3.534 (3) Å], and weak inter­molecular C—H⋯O and C—H⋯Cl hydrogen bonds.

## Related literature

The simple preparation of 2-chloro-1-ferrocenyl­ethanone was described previously by Ferreira *et al.* (2009 ▶). For the crystal structures of ferrocenyl complexes of the type [FeCp(C_5_H_4_CO*R*)], where Cp is η^5^-C_5_H_5_ and *R* is CH_3_ or CH_2_I, see: Sato *et al.* (1984 ▶); Khrustalev *et al.* (2006 ▶); McAdam *et al.* (2006 ▶). For the use of acyl­ferrocenes as catalysts for the autoxidation of alkyd resins, see: Štáva *et al.* (2007 ▶); Kalenda *et al.* (2010 ▶).
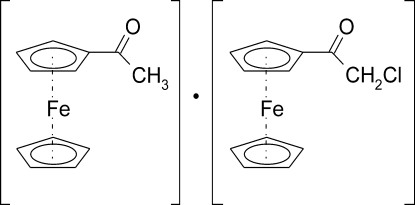


## Experimental

### 

#### Crystal data

[Fe(C_5_H_5_)(C_7_H_6_ClO)][Fe(C_5_H_5_)(C_7_H_7_O)]
*M* *_r_* = 490.56
Monoclinic, 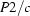

*a* = 15.2981 (11) Å
*b* = 5.7338 (3) Å
*c* = 24.4051 (12) Å
β = 112.031 (7)°
*V* = 1984.5 (2) Å^3^
*Z* = 4
Mo *K*α radiation
μ = 1.62 mm^−1^
*T* = 150 K
0.15 × 0.10 × 0.08 mm

#### Data collection

Nonius KappaCCD area-detector diffractometer
Absorption correction: gaussian (Coppens, 1970 ▶) *T*_min_ = 0.791, *T*_max_ = 0.886
9948 measured reflections
4502 independent reflections
3232 reflections with *I* > 2σ(*I*)
*R*_int_ = 0.052

#### Refinement

*R*[*F* ^2^ > 2σ(*F* ^2^)] = 0.059
*wR*(*F* ^2^) = 0.151
*S* = 0.97
4502 reflections
262 parameters
H-atom parameters constrained
Δρ_max_ = 0.48 e Å^−3^
Δρ_min_ = −1.22 e Å^−3^

### 

Data collection: *COLLECT* (Hooft, 1998 ▶); cell refinement: *DENZO*/*SCALEPACK* (Otwinowski & Minor, 1997 ▶); data reduction: *DENZO*/*SCALEPACK*; program(s) used to solve structure: *SIR92* (Altomare *et al.*, 1994 ▶); program(s) used to refine structure: *SHELXL97* (Sheldrick, 2008 ▶); molecular graphics: *PLATON* (Spek, 2009 ▶); software used to prepare material for publication: *enCIFer* (Allen *et al.*, 2004 ▶).

## Supplementary Material

Crystal structure: contains datablock(s) global, I. DOI: 10.1107/S1600536811038244/cv5144sup1.cif

Structure factors: contains datablock(s) I. DOI: 10.1107/S1600536811038244/cv5144Isup2.hkl

Additional supplementary materials:  crystallographic information; 3D view; checkCIF report

## Figures and Tables

**Table 1:** Hydrogen-bond geometry (Å, °)

*D*—H⋯*A*	*D*—H	H⋯*A*	*D*⋯*A*	*D*—H⋯*A*
C4—H4⋯O2^i^	0.93	2.51	3.333 (6)	148
C14—H14⋯O2^ii^	0.93	2.63	3.424 (6)	144
C20—H20⋯Cl1	0.93	2.76	3.602 (8)	152

Symmetry codes: (i) 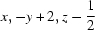; (ii) 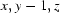.

**Table 2:** Selected geometric parameters (Å, °) *Cg*1, *Cg*2, *Cg*3 and *Cg*4 are the centroids of the C1–C5, C8–C12, C13–C17 and C20–C24 rings, respectively.

2-Chloro-1-ferrocenyl­ethanone		Acetyl­ferrocene	
Fe1⋯*Cg*1	1.643 (1)	Fe2⋯*Cg*3	1.646 (2)
Fe1⋯*Cg*2	1.648 (2)	Fe2⋯*Cg*4	1.653 (3)
*Cg*1⋯Fe1⋯*Cg*2	178.06 (11)	*Cg*3⋯Fe2⋯*Cg*4	179.11 (15)

**Fig. 1.**
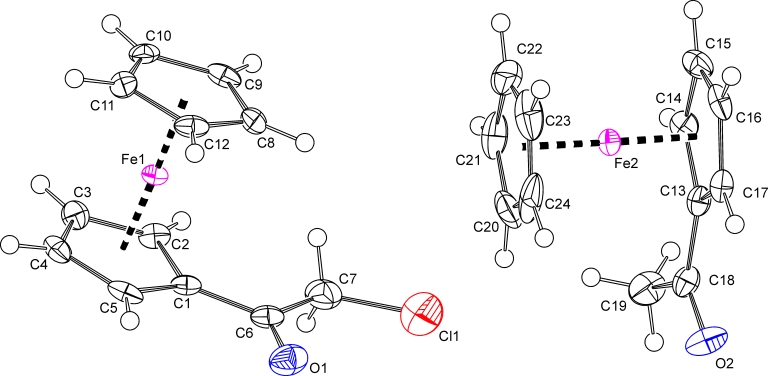
A content of asymmetric part of (I), showing 50% probability displacement ellipsoids and the atom-numbering scheme.

## supplementary crystallographic information

### Comment

Substituted ferrocenes belong to well known class of organometallic compounds that are currently studied as catalysts, in drug design, as building blocks in material engineering or in nanotechnology. Recently, we have shown that ferrocene complexes bearing electron-withdrawing acyl substituents at the cyclopentadienyl (Cp) ring could be used as driers for autoxidation of alkyd resins (Štáva *et al.*, 2007; Kalenda *et al.*, 2010). During our investigation of drying activity of ferrocene derivatives we prepared various stock solutions containing a mixture of acylferrocenes. From the mixture of acetylferrocene and 2-chloro-1-ferrocenylethanone in cyclohexane crystals of the title compound (I) were grown. Herewith we present crystal structure of (I).

The asymmetric unit of (I) contains one molecule of acetylferrocene and one molecule of 2-chloro-1-ferrocenylethanone. Molecule of acetylferrocene has geometrical parameters very close to that reported for acetylferrocene by Sato *et al.* (1984) and by Khrustalev *et al.* (2006), see Table 1.

2-Chloro-1-ferrocenylethanone has a structure typical for monosubstituted ferrocene with almost eclipsed Cp rings. The dihedral angle C1—*Cg*1—*Cg*2—C8 was found to be 2.2 (4)°. Carbonyl *sp*^2^ atom C6 is slightly displaced from the Cp ring plane toward the Fe atom, with an angle of 3.0 (3)° between C1—C6 bond and the ring plane. The shortening of single bond length C1—C6 to a value of 1.459 (7) Å together with elongation of double bond C6═O1 [1.221 (6) Å] indicate significant conjugation of carbonyl substituent with adjacent Cp ring π-system. These values are similar to those observed for 2-iodo-1-ferrocenylethanone (McAdam *et al.*, 2006).

Substituted Cp ring of molecule 2-chloro-1-ferrocenylethanone at (*x*, *y*, *z*) is coplanar with substituted Cp ring of molecule at (-*x*, 2 - *y*, -*z*) with the distance between the centroids of 3.534 (3) Å. Thus molecules of 2-chloro-1-ferrocenylethanone form pairs due to π···π stacking. Simultaneously, the molecule at (*x*, *y*, *z*) show C—Cl···Cl—C contact to the molecule at (-*x*, *y*, ^1^/_2_-*z*) with the Cl···Cl distance of 3.279 (4) Å giving infinite wires of 2-chloro-1-ferrocenylethanone molecules along the *c* axis. Molecular wires of 2-chloro-1-ferrocenylethanone are connected with molecules of acetylferrocene *via* weak C—H···O2 and C—H···Cl1 hydrogen bonds (Table 2) giving observed three-dimensional structure.

### Experimental

Red crystals of (I) suitable for X-ray diffraction analysis were grown by slow evaporation of cyclohexane solution containing acetylferrocene (purchased from Sigma Aldrich) and 2-chloro-1-ferrocenylethanone in 1:1 molar ratio. The 2-chloro-1-ferrocenylethanone has been prepared from ferrocene and chloroacetyl chloride following method of Ferreira *et al.* (2009).

### Crystal data

**Table:**

[Fe(C_5_H_5_)(C_7_H_6_ClO)][Fe(C_5_H_5_)(C_7_H_7_O)]	*F*(000) = 1008
*M**_r_* = 490.56	*D*_x_ = 1.642 Mg m^−^^3^
Monoclinic, *P*2/*c*	Melting point: 350 K
Hall symbol: -P 2yc	Mo *K*α radiation, λ = 0.71073 Å
*a* = 15.2981 (11) Å	Cell parameters from 9981 reflections
*b* = 5.7338 (3) Å	θ = 1–27.5°
*c* = 24.4051 (12) Å	µ = 1.62 mm^−^^1^
β = 112.031 (7)°	*T* = 150 K
*V* = 1984.5 (2) Å^3^	Block, red
*Z* = 4	0.15 × 0.10 × 0.08 mm

### Data collection

**Table:**

Nonius KappaCCD area-detector diffractometer	4502 independent reflections
Radiation source: fine-focus sealed tube	3232 reflections with *I* > 2σ(*I*)
graphite	*R*_int_ = 0.052
Detector resolution: 9.091 pixels mm^-1^	θ_max_ = 27.5°, θ_min_ = 2.7°
φ and ω scans to fill the Ewald sphere	*h* = −18→19
Absorption correction: gaussian (Coppens, 1970)	*k* = −5→7
*T*_min_ = 0.791, *T*_max_ = 0.886	*l* = −21→31
9948 measured reflections	

### Refinement

**Table:**

Refinement on *F*^2^	Primary atom site location: structure-invariant direct methods
Least-squares matrix: full	Secondary atom site location: difference Fourier map
*R*[*F*^2^ > 2σ(*F*^2^)] = 0.059	Hydrogen site location: inferred from neighbouring sites
*wR*(*F*^2^) = 0.151	H-atom parameters constrained
*S* = 0.97	*w* = 1/[σ^2^(*F*_o_^2^) + (0.063*P*)^2^ + 9.3142*P*] where *P* = (*F*_o_^2^ + 2*F*_c_^2^)/3
4502 reflections	(Δ/σ)_max_ < 0.001
262 parameters	Δρ_max_ = 0.48 e Å^−^^3^
0 restraints	Δρ_min_ = −1.22 e Å^−^^3^

### Special details

**Table:**

Experimental. Melting point: 349–350 K. Spectroscopic analysis: IR (diamond ATR, cm^-1^): 3097 (*m*), 2955 (*m*), 2916 (*s*), 2848 (*s*), 1673 (*m*), 1660 (m, sh), 1652 (*versus*), 1451 (*m*), 1408 (w), 1374 (*s*), 1356 (*m*), 1278 (*versus*), 1240 (*m*), 1103 (*m*), 1066 (w), 1038 (*m*), 960 (w), 892 (*m*), 848 (w), 819 (*versus*), 720 (*m*), 668 (w), 618 (*s*), 593 (w), 531 (*s*), 494 (*s*), 480 (*versus*), 458(*s*).
Geometry. All e.s.d.'s (except the e.s.d. in the dihedral angle between two l.s. planes) are estimated using the full covariance matrix. The cell e.s.d.'s are taken into account individually in the estimation of e.s.d.'s in distances, angles and torsion angles; correlations between e.s.d.'s in cell parameters are only used when they are defined by crystal symmetry. An approximate (isotropic) treatment of cell e.s.d.'s is used for estimating e.s.d.'s involving l.s. planes.
Refinement. Refinement of *F*^2^ against ALL reflections. The weighted *R*-factor *wR* and goodness of fit *S* are based on *F*^2^, conventional *R*-factors *R* are based on *F*, with *F* set to zero for negative *F*^2^. The threshold expression of *F*^2^ > σ(*F*^2^) is used only for calculating *R*-factors(gt) *etc*. and is not relevant to the choice of reflections for refinement. *R*-factors based on *F*^2^ are statistically about twice as large as those based on *F*, and *R*- factors based on ALL data will be even larger.

### Fractional atomic coordinates and isotropic or equivalent isotropic displacement parameters (Å^2^)

**Table:**

	*x*	*y*	*z*	*U*_iso_*/*U*_eq_	
Fe1	0.22439 (4)	0.81308 (10)	0.05785 (3)	0.01538 (18)	
Fe2	0.36043 (5)	0.35801 (11)	0.34458 (3)	0.01683 (18)	
Cl1	0.08171 (16)	0.8811 (4)	0.22170 (9)	0.0682 (6)	
O1	0.1117 (3)	1.2154 (6)	0.13777 (18)	0.0347 (9)	
C10	0.3214 (3)	0.5641 (8)	0.0624 (2)	0.0215 (10)	
H10	0.3124	0.4388	0.0367	0.026*	
C1	0.1050 (3)	0.9508 (8)	0.0629 (2)	0.0199 (10)	
C2	0.0869 (3)	0.7242 (8)	0.0358 (2)	0.0195 (10)	
H2	0.0652	0.5943	0.0497	0.023*	
O2	0.1710 (3)	0.6485 (6)	0.38637 (19)	0.0358 (9)	
C12	0.3591 (3)	0.9268 (8)	0.1048 (2)	0.0228 (10)	
H12	0.3785	1.0814	0.1117	0.027*	
C14	0.3080 (3)	0.1198 (8)	0.3857 (2)	0.0222 (10)	
H14	0.2677	−0.0025	0.3674	0.027*	
C18	0.1843 (3)	0.4500 (9)	0.3721 (2)	0.0238 (10)	
C3	0.1083 (3)	0.7360 (9)	−0.0156 (2)	0.0231 (10)	
H3	0.1021	0.6144	−0.0421	0.028*	
C13	0.2802 (3)	0.3504 (8)	0.3943 (2)	0.0198 (10)	
C11	0.3566 (3)	0.7881 (9)	0.0558 (2)	0.0211 (10)	
H11	0.3748	0.8349	0.0251	0.025*	
C15	0.4080 (4)	0.1097 (9)	0.4101 (2)	0.0259 (11)	
H15	0.4448	−0.0204	0.4110	0.031*	
C5	0.1390 (3)	1.0972 (8)	0.0278 (2)	0.0226 (10)	
H5	0.1564	1.2530	0.0352	0.027*	
C9	0.3028 (3)	0.5646 (8)	0.1146 (2)	0.0235 (11)	
H9	0.2792	0.4401	0.1293	0.028*	
C17	0.3638 (3)	0.4823 (8)	0.4241 (2)	0.0204 (10)	
H17	0.3666	0.6379	0.4354	0.024*	
C8	0.3258 (3)	0.7881 (9)	0.1412 (2)	0.0238 (11)	
H8	0.3204	0.8349	0.1763	0.029*	
C4	0.1408 (3)	0.9647 (9)	−0.0203 (2)	0.0228 (10)	
H4	0.1597	1.0180	−0.0501	0.027*	
C16	0.4420 (4)	0.3345 (9)	0.4331 (2)	0.0248 (11)	
H16	0.5050	0.3769	0.4510	0.030*	
C7	0.0684 (4)	0.8231 (10)	0.1510 (3)	0.0348 (13)	
H7A	0.1049	0.6851	0.1507	0.042*	
H7B	0.0027	0.7871	0.1286	0.042*	
C23	0.4422 (4)	0.4927 (12)	0.3029 (2)	0.0408 (16)	
H23	0.5052	0.5363	0.3207	0.049*	
C21	0.3125 (4)	0.2756 (11)	0.2570 (2)	0.0382 (14)	
H21	0.2738	0.1502	0.2391	0.046*	
C6	0.0974 (3)	1.0163 (9)	0.1187 (2)	0.0234 (10)	
C19	0.1050 (4)	0.3049 (10)	0.3315 (3)	0.0340 (13)	
H19A	0.0461	0.3740	0.3282	0.041*	
H19B	0.1087	0.1491	0.3466	0.041*	
H19C	0.1091	0.3003	0.2932	0.041*	
C24	0.3630 (7)	0.6347 (10)	0.2938 (3)	0.057 (2)	
H24	0.3632	0.7901	0.3047	0.068*	
C22	0.4093 (4)	0.2720 (11)	0.2798 (3)	0.0372 (13)	
H22	0.4464	0.1436	0.2799	0.045*	
C20	0.2817 (5)	0.4942 (13)	0.2643 (3)	0.0453 (17)	
H20	0.2192	0.5413	0.2526	0.054*	

### Atomic displacement parameters (Å^2^)

**Table:**

	*U*^11^	*U*^22^	*U*^33^	*U*^12^	*U*^13^	*U*^23^
Fe1	0.0120 (3)	0.0136 (3)	0.0193 (4)	0.0013 (2)	0.0044 (3)	0.0008 (3)
Fe2	0.0177 (3)	0.0177 (3)	0.0147 (3)	−0.0025 (3)	0.0056 (3)	−0.0002 (3)
Cl1	0.0756 (14)	0.0759 (13)	0.0513 (11)	0.0204 (11)	0.0220 (10)	−0.0051 (10)
O1	0.031 (2)	0.030 (2)	0.040 (2)	0.0007 (16)	0.0092 (17)	−0.0108 (18)
C10	0.014 (2)	0.023 (2)	0.025 (3)	0.0067 (18)	0.0044 (19)	−0.003 (2)
C1	0.013 (2)	0.019 (2)	0.026 (3)	0.0046 (18)	0.0057 (19)	0.001 (2)
C2	0.012 (2)	0.017 (2)	0.029 (3)	−0.0045 (17)	0.0077 (19)	−0.002 (2)
O2	0.033 (2)	0.027 (2)	0.054 (3)	0.0020 (16)	0.024 (2)	−0.0088 (18)
C12	0.012 (2)	0.019 (2)	0.032 (3)	0.0011 (18)	0.003 (2)	−0.003 (2)
C14	0.025 (3)	0.018 (2)	0.025 (2)	−0.0031 (19)	0.011 (2)	0.0023 (19)
C18	0.023 (2)	0.028 (3)	0.025 (3)	−0.004 (2)	0.015 (2)	−0.001 (2)
C3	0.020 (2)	0.026 (2)	0.020 (2)	−0.001 (2)	0.0035 (19)	−0.002 (2)
C13	0.023 (2)	0.020 (2)	0.018 (2)	−0.0055 (19)	0.0099 (19)	−0.0015 (19)
C11	0.013 (2)	0.030 (3)	0.023 (2)	0.0003 (19)	0.0094 (19)	0.002 (2)
C15	0.025 (3)	0.024 (3)	0.028 (3)	0.002 (2)	0.009 (2)	0.009 (2)
C5	0.013 (2)	0.016 (2)	0.033 (3)	0.0019 (17)	0.002 (2)	0.008 (2)
C9	0.013 (2)	0.020 (2)	0.032 (3)	0.0011 (18)	0.003 (2)	0.008 (2)
C17	0.028 (3)	0.018 (2)	0.017 (2)	−0.0087 (19)	0.010 (2)	−0.0028 (19)
C8	0.016 (2)	0.034 (3)	0.016 (2)	0.006 (2)	0.0000 (19)	0.001 (2)
C4	0.017 (2)	0.026 (2)	0.021 (2)	0.0020 (19)	0.003 (2)	0.007 (2)
C16	0.022 (2)	0.033 (3)	0.015 (2)	−0.007 (2)	0.0019 (19)	0.006 (2)
C7	0.024 (3)	0.041 (3)	0.039 (3)	0.007 (2)	0.011 (2)	0.005 (3)
C23	0.031 (3)	0.066 (4)	0.025 (3)	−0.024 (3)	0.010 (2)	0.006 (3)
C21	0.049 (4)	0.046 (3)	0.021 (3)	−0.019 (3)	0.014 (3)	−0.010 (3)
C6	0.012 (2)	0.025 (3)	0.031 (3)	0.0035 (19)	0.006 (2)	0.000 (2)
C19	0.023 (3)	0.039 (3)	0.037 (3)	−0.004 (2)	0.008 (2)	−0.010 (3)
C24	0.138 (8)	0.018 (3)	0.034 (3)	0.001 (4)	0.055 (4)	0.004 (3)
C22	0.044 (3)	0.042 (3)	0.036 (3)	0.006 (3)	0.027 (3)	0.006 (3)
C20	0.039 (3)	0.073 (5)	0.025 (3)	0.019 (3)	0.013 (3)	0.025 (3)

### Geometric parameters (Å, °)

**Table:**

Fe1—C10	2.032 (5)	C14—C13	1.428 (6)
Fe1—C2	2.031 (4)	C14—H14	0.9300
Fe1—C9	2.035 (5)	C18—C13	1.474 (7)
Fe1—C1	2.036 (4)	C18—C19	1.499 (7)
Fe1—C8	2.048 (5)	C3—C4	1.422 (7)
Fe1—C5	2.044 (5)	C3—H3	0.9301
Fe1—C3	2.043 (5)	C13—C17	1.428 (6)
Fe1—C12	2.053 (5)	C11—H11	0.9299
Fe1—C11	2.047 (4)	C15—C16	1.424 (7)
Fe1—C4	2.050 (5)	C15—H15	0.9300
Fe2—C20	2.032 (6)	C5—C4	1.407 (7)
Fe2—C24	2.023 (6)	C5—H5	0.9300
Fe2—C13	2.025 (5)	C9—C8	1.420 (7)
Fe2—C21	2.037 (5)	C9—H9	0.9301
Fe2—C14	2.031 (5)	C17—C16	1.414 (7)
Fe2—C23	2.038 (5)	C17—H17	0.9300
Fe2—C22	2.046 (5)	C8—H8	0.9300
Fe2—C17	2.049 (5)	C4—H4	0.9299
Fe2—C16	2.055 (5)	C16—H16	0.9299
Fe2—C15	2.058 (5)	C7—C6	1.519 (7)
Cl1—C7	1.693 (6)	C7—H7A	0.9699
O1—C6	1.222 (6)	C7—H7B	0.9700
C10—C9	1.405 (7)	C23—C22	1.400 (9)
C10—C11	1.426 (7)	C23—C24	1.407 (10)
C10—H10	0.9299	C23—H23	0.9300
C1—C5	1.430 (7)	C21—C22	1.372 (8)
C1—C2	1.437 (6)	C21—C20	1.374 (9)
C1—C6	1.458 (7)	C21—H21	0.9300
C2—C3	1.413 (7)	C19—H19A	0.9600
C2—H2	0.9299	C19—H19B	0.9600
O2—C18	1.229 (6)	C19—H19C	0.9601
C12—C8	1.421 (7)	C24—C20	1.429 (10)
C12—C11	1.426 (7)	C24—H24	0.9300
C12—H12	0.9300	C22—H22	0.9300
C14—C15	1.419 (7)	C20—H20	0.9300
			
C10—Fe1—C2	120.04 (19)	C15—C14—C13	108.1 (4)
C10—Fe1—C9	40.4 (2)	C15—C14—Fe2	70.7 (3)
C2—Fe1—C9	106.99 (19)	C13—C14—Fe2	69.1 (3)
C10—Fe1—C1	157.31 (19)	C15—C14—H14	125.9
C2—Fe1—C1	41.38 (19)	C13—C14—H14	126.1
C9—Fe1—C1	122.9 (2)	Fe2—C14—H14	125.5
C10—Fe1—C8	68.3 (2)	O2—C18—C13	120.2 (5)
C2—Fe1—C8	124.7 (2)	O2—C18—C19	121.5 (5)
C9—Fe1—C8	40.7 (2)	C13—C18—C19	118.3 (4)
C1—Fe1—C8	109.2 (2)	C4—C3—C2	108.7 (4)
C10—Fe1—C5	159.2 (2)	C4—C3—Fe1	69.9 (3)
C2—Fe1—C5	69.28 (18)	C2—C3—Fe1	69.3 (3)
C9—Fe1—C5	159.4 (2)	C4—C3—H3	125.7
C1—Fe1—C5	41.03 (19)	C2—C3—H3	125.7
C8—Fe1—C5	123.8 (2)	Fe1—C3—H3	126.8
C10—Fe1—C3	105.8 (2)	C17—C13—C14	107.8 (4)
C2—Fe1—C3	40.6 (2)	C17—C13—C18	124.0 (4)
C9—Fe1—C3	122.8 (2)	C14—C13—C18	127.9 (4)
C1—Fe1—C3	68.5 (2)	C17—C13—Fe2	70.4 (3)
C8—Fe1—C3	160.3 (2)	C14—C13—Fe2	69.6 (3)
C5—Fe1—C3	68.3 (2)	C18—C13—Fe2	121.0 (3)
C10—Fe1—C12	68.47 (19)	C12—C11—C10	107.4 (4)
C2—Fe1—C12	161.9 (2)	C12—C11—Fe1	69.9 (3)
C9—Fe1—C12	68.31 (19)	C10—C11—Fe1	69.0 (2)
C1—Fe1—C12	125.36 (19)	C12—C11—H11	126.3
C8—Fe1—C12	40.6 (2)	C10—C11—H11	126.3
C5—Fe1—C12	108.64 (19)	Fe1—C11—H11	126.6
C3—Fe1—C12	156.7 (2)	C14—C15—C16	107.8 (4)
C10—Fe1—C11	40.9 (2)	C14—C15—Fe2	68.7 (3)
C2—Fe1—C11	155.59 (19)	C16—C15—Fe2	69.6 (3)
C9—Fe1—C11	68.43 (19)	C14—C15—H15	126.2
C1—Fe1—C11	161.07 (19)	C16—C15—H15	126.0
C8—Fe1—C11	68.4 (2)	Fe2—C15—H15	127.4
C5—Fe1—C11	123.43 (19)	C4—C5—C1	107.9 (4)
C3—Fe1—C11	120.3 (2)	C4—C5—Fe1	70.1 (3)
C12—Fe1—C11	40.69 (19)	C1—C5—Fe1	69.2 (2)
C10—Fe1—C4	122.5 (2)	C4—C5—H5	126.0
C2—Fe1—C4	68.71 (19)	C1—C5—H5	126.1
C9—Fe1—C4	158.9 (2)	Fe1—C5—H5	126.8
C1—Fe1—C4	68.33 (19)	C10—C9—C8	108.4 (4)
C8—Fe1—C4	158.4 (2)	C10—C9—Fe1	69.7 (3)
C5—Fe1—C4	40.2 (2)	C8—C9—Fe1	70.2 (3)
C3—Fe1—C4	40.65 (19)	C10—C9—H9	125.9
C12—Fe1—C4	122.0 (2)	C8—C9—H9	125.6
C11—Fe1—C4	106.5 (2)	Fe1—C9—H9	125.8
C20—Fe2—C24	41.3 (3)	C16—C17—C13	107.8 (4)
C20—Fe2—C13	108.2 (2)	C16—C17—Fe2	70.1 (3)
C24—Fe2—C13	122.7 (3)	C13—C17—Fe2	68.5 (3)
C20—Fe2—C21	39.5 (3)	C16—C17—H17	126.0
C24—Fe2—C21	67.7 (3)	C13—C17—H17	126.2
C13—Fe2—C21	124.3 (2)	Fe2—C17—H17	126.6
C20—Fe2—C14	122.3 (2)	C9—C8—C12	107.8 (4)
C24—Fe2—C14	158.9 (3)	C9—C8—Fe1	69.2 (3)
C13—Fe2—C14	41.24 (19)	C12—C8—Fe1	69.9 (3)
C21—Fe2—C14	108.0 (2)	C9—C8—H8	126.1
C20—Fe2—C23	68.0 (3)	C12—C8—H8	126.1
C24—Fe2—C23	40.5 (3)	Fe1—C8—H8	126.8
C13—Fe2—C23	158.7 (2)	C5—C4—C3	108.3 (4)
C21—Fe2—C23	67.1 (2)	C5—C4—Fe1	69.7 (3)
C14—Fe2—C23	158.8 (3)	C3—C4—Fe1	69.4 (3)
C20—Fe2—C22	66.6 (3)	C5—C4—H4	125.7
C24—Fe2—C22	67.6 (3)	C3—C4—H4	126.0
C13—Fe2—C22	159.6 (2)	Fe1—C4—H4	126.8
C21—Fe2—C22	39.3 (2)	C17—C16—C15	108.6 (4)
C14—Fe2—C22	123.1 (2)	C17—C16—Fe2	69.6 (3)
C23—Fe2—C22	40.1 (3)	C15—C16—Fe2	69.9 (3)
C20—Fe2—C17	125.1 (2)	C17—C16—H16	125.8
C24—Fe2—C17	107.9 (2)	C15—C16—H16	125.6
C13—Fe2—C17	41.04 (18)	Fe2—C16—H16	126.1
C21—Fe2—C17	160.9 (2)	C6—C7—Cl1	116.1 (4)
C14—Fe2—C17	68.89 (19)	C6—C7—H7A	108.1
C23—Fe2—C17	122.6 (2)	Cl1—C7—H7A	108.6
C22—Fe2—C17	158.2 (2)	C6—C7—H7B	107.8
C20—Fe2—C16	161.0 (3)	Cl1—C7—H7B	108.6
C24—Fe2—C16	123.5 (3)	H7A—C7—H7B	107.5
C13—Fe2—C16	68.5 (2)	C22—C23—C24	107.5 (5)
C21—Fe2—C16	157.7 (2)	C22—C23—Fe2	70.2 (3)
C14—Fe2—C16	68.4 (2)	C24—C23—Fe2	69.1 (3)
C23—Fe2—C16	107.8 (2)	C22—C23—H23	125.8
C22—Fe2—C16	122.9 (2)	C24—C23—H23	126.8
C17—Fe2—C16	40.3 (2)	Fe2—C23—H23	126.2
C20—Fe2—C15	157.4 (3)	C22—C21—C20	109.4 (6)
C24—Fe2—C15	159.4 (3)	C22—C21—Fe2	70.7 (3)
C13—Fe2—C15	68.7 (2)	C20—C21—Fe2	70.1 (3)
C21—Fe2—C15	122.5 (2)	C22—C21—H21	125.4
C14—Fe2—C15	40.61 (19)	C20—C21—H21	125.3
C23—Fe2—C15	123.1 (2)	Fe2—C21—H21	126.0
C22—Fe2—C15	107.9 (2)	O1—C6—C1	122.1 (5)
C17—Fe2—C15	68.2 (2)	O1—C6—C7	121.9 (5)
C16—Fe2—C15	40.5 (2)	C1—C6—C7	116.0 (4)
C9—C10—C11	108.4 (4)	C18—C19—H19A	109.1
C9—C10—Fe1	69.9 (3)	C18—C19—H19B	110.3
C11—C10—Fe1	70.1 (3)	H19A—C19—H19B	109.5
C9—C10—H10	125.8	C18—C19—H19C	109.1
C11—C10—H10	125.8	H19A—C19—H19C	109.5
Fe1—C10—H10	125.9	H19B—C19—H19C	109.5
C5—C1—C2	107.8 (4)	C20—C24—C23	106.8 (5)
C5—C1—C6	125.6 (4)	C20—C24—Fe2	69.7 (3)
C2—C1—C6	126.4 (4)	C23—C24—Fe2	70.3 (3)
C5—C1—Fe1	69.8 (3)	C20—C24—H24	126.4
C2—C1—Fe1	69.1 (2)	C23—C24—H24	126.8
C6—C1—Fe1	122.9 (3)	Fe2—C24—H24	125.0
C3—C2—C1	107.3 (4)	C21—C22—C23	108.7 (6)
C3—C2—Fe1	70.2 (3)	C21—C22—Fe2	70.0 (3)
C1—C2—Fe1	69.5 (2)	C23—C22—Fe2	69.7 (3)
C3—C2—H2	126.6	C21—C22—H22	125.3
C1—C2—H2	126.1	C23—C22—H22	126.0
Fe1—C2—H2	125.5	Fe2—C22—H22	126.1
C8—C12—C11	108.0 (4)	C21—C20—C24	107.7 (6)
C8—C12—Fe1	69.5 (3)	C21—C20—Fe2	70.5 (3)
C11—C12—Fe1	69.4 (3)	C24—C20—Fe2	69.0 (3)
C8—C12—H12	125.8	C21—C20—H20	126.1
C11—C12—H12	126.1	C24—C20—H20	126.3
Fe1—C12—H12	126.1	Fe2—C20—H20	125.2

### Hydrogen-bond geometry (Å, °)

**Table:**

*D*—H···*A*	*D*—H	H···*A*	*D*···*A*	*D*—H···*A*
C4—H4···O2^i^	0.93	2.51	3.333 (6)	148
C14—H14···O2^ii^	0.93	2.63	3.424 (6)	144
C20—H20···Cl1	0.93	2.76	3.602 (8)	152

Symmetry codes: (i) *x*, −*y*+2, *z*−1/2; (ii) *x*, *y*−1, *z*.

### Table 2 Selected geometric parameters (Å, °).

**Table:**

2-Chloro-1-ferrocenylethanone		Acetylferrocene	
Fe1···Cg1	1.643 (1)	Fe2···Cg3	1.646 (2)
Fe1···Cg2	1.648 (2)	Fe2···Cg4	1.653 (3)
Cg1···Fe1···Cg2	178.06 (11)	Cg3···Fe2···Cg4	179.11 (15)

Cg1, Cg2, Cg3 and Cg4 are the centroids of the C1–C5, C8–C12, C13–C17 and C20–C24 rings, respectively.
